# Low levels of vitamin D were associated with coagulopathy among hospitalized coronavirus disease-19 (COVID-19) patients: A single-centered study in Indonesia

**DOI:** 10.5937/jomb0-30228

**Published:** 2021-09-03

**Authors:** Hani Susianti, Cesarius Singgih Wahono, Perdana Aditya Rahman, Mirza Zaka Pratama, Indah Adhita Wulanda, Khoirunisah Dwi Hartanti, Elvira Sari Dewi, Kusworini Handono

**Affiliations:** 1 University of Brawijaya, Faculty of Medicine, Department of Clinical Pathology, Malang, Indonesia; 2 University of Brawijaya, Faculty of Medicine, Department of Internal Medicine, Rheumatology and Immunology Division, Malang, Indonesia; 3 University of Brawijaya, Faculty of Medicine, Basic Nursing Department, Malang, Indonesia

**Keywords:** vitamin D, COVID-19, coagulopathy, vitamin D, COVID-19, koagulopatija

## Abstract

**Background:**

This study was aimed to explore the association of vitamin D in the risk of coagulopathy in coronavirus disease-19 (COVID-19).

**Methods:**

Clinical and laboratory findings were obtained from 50 confirmed COVID-19 patients hospitalized in Saiful Anwar General Hospital, Malang, Indonesia, from September to November 2020. Thrombotic events during hospitalization were recorded, and the ISTH disseminated intravascular coagulation (DIC) score was used to classify overt DIC. Hypovitaminosis D was defined by serum vitamin D level <49.92 nmol/L.

**Results:**

Among 50 patients, 42 (84%) had hypovitaminosis D, and 6 (12%) developed thrombotic events. Vitamin D levels were lower in patients with thrombotic events (p=0.015), D-dimer >2 mg/L (p=0.006), ISTH DIC score 5 (p=0.020), admitted on ICU (p=0.002), and non-survivor groups (p=0.007). Multivariate analysis for the risk in increased D-dimer levels showed low vitamin D as the only significant risk factor with OR 1.8 (1.2-4.4), p=0.034. Low vitamin D also increased the risk for developing overt DIC with OR. 5.4 (1.0-30.2), p=0.039. Vitamin D level had negative correlations with ferritin (R=-0.316, p=0.044) and CRP (R=-0.530, p=0.000).

**Conclusions:**

In conclusion, a low level of vitamin D was found in most hospitalized COVID-19 patients and might be associated with the development of coagulopathy.

## Abstract (sr)

**Uvod:**

Cilj ove studije je bio da istraži povezanost vitamina D sa rizikom od koagulopatije kod koronavirusne bolesti 2019 (COVID-19).

**Metode:**

Klinički i laboratorijski nalazi dobijeni su od 50 potvrđenih pacijenata sa COVID-19 hospitalizovanih u opštoj bolnici Saiful Anvar u Malangu u Indoneziji, od septembra do novembra 2020. Zabeleženi su trombotični događaji tokom hospitalizacije, a rezultati ISTH diseminovane intravaskularne koagulacije (DIC) korišćeni su za klasifikaciju očigledne DIC. Hipovitaminoza D je definisana nivoom vitamina D u serumu <49,92 nmol/L.

**Rezultati:**

Od 50 pacijenata, 42 (84%) je imalo hipovitaminozu D, a 6 (12%) je razvilo trombotičke događaje. Nivoi vitamina D su bili niži kod pacijenata sa trombotičkim događajima (p=0,015), D-dimerom >2 mg/L (p=0,006), ISTH DIC rezultatima 5 (p=0,020), primljenih na intenzivnu negu (p=0,002), i grupa koje nisu preživele (p=0,007). Multivarijantna analiza rizika za povećane nivoe D-dimera pokazala je nizak nivo vitamina D kao jedini značajni faktor rizika sa OR 1,8 (1,2-4,4), p=0,034. Nizak nivo vitamina D je takođe povećao rizik od razvoja očiglednog DIC-a sa OR 5,4 (1,0-30,2), p=0,039. Nivo vitamina D je imao negativne korelacije sa feritinom (R=-0,316, p=0,044) i CRP-om (R=-0,530, p=0,000).

**Zaključak:**

Na kraju, kod većine hospitalizovanih pacijenata sa COVID-19 utvrđen je nizak nivo vitamina D koji bi mogao biti povezan sa razvojem koagulopatije.

## Introduction

The novel coronavirus, severe acute respiratory syndrome coronavirus 2 (SARS-Cov-2) emerged first in Wuhan, China, at the end of 2019 and caused pandemic that is still ongoing. It causes a disease called coronavirus disease 2019 (COVID-19) and has affected more than 60 million people worldwide until November 2020 [1]. Reported cases of COVID-19 show that this disease ranges from asymptomatic or mild infection to multi-organ failure and death [2]. COVID-19 is a systemic disease that affects not only the respiratory tract but also other system organs. Coagulopathy is one of the most common complications reported in several studies, which can be manifested as venous or arterial thromboembolism [3]. A marker of a fibrinogen degradation product, D-dimer, also increases in 3.75-68.0% of COVID-19 patients and elevates the D-dimer level >2 mg/L at admission which predicts more severe and higher mortality of the disease [4] [5].

International Society of Thrombosis and Hemostasis (ISTH) develops a new guideline for managing coagulopathy in COVID-19. Anticoagulant prophylaxis should be given to all COVID-19 patients with markedly increased coagulation markers, including D-dimer [6]. The coagulopathy mechanism in COVID-19 has not been established yet. However, it may overlap with bacteria-induced septic coagulopathy or disseminated intravascular coagulation (DIC). Proinflammatory conditions in COVID-19 through cytokine storm, complement activation, increased damage-associated molecular pattern, and vascular endothelial damage is significant factors that lead to thrombosis [7] [8]. A previous study also showed that this novel coronavirus could also induce secondary anti-phospholipid syndrome (APS) by promoting the production of anti-phospholipid antibodies (APL-Abs) [9]. Xiao et al. [10] also support this study, which found that 47% of patients with COVID-19 in the intensive unit were positive for APL-Abs. However, it is still unclear whether these antibodies are associated with thrombosis in these patients.

Another novel proposed mechanisms of coagulopathy in COVID-19 is the role of vitamin D in the coagulation system [11]. The effect of vitamin D metabolites through vitamin D receptor (VDR) ligands can be attributed to the anti-thrombotic effects. VDR knockout mice have been shown to have a significant association with the presence of thrombosis in several organs [12]. Moreover, Khamdevatani et al. [13]. have described that a low level of vitamin D is associated with lower limb deep vein thrombosis (DVT). The role of vitamin D has already been mentioned before in COVID-19 [14] [15] but rarely reported in COVID-19 coagulopathy. Therefore, this study was aimed to examine the association of serum vitamin D levels in the presence of COVID-19 coagulopathy. This study also compared the role of vitamin D in coagulopathy with other surrogate markers reported previously associated with COVID-19 severity, such as ferritin, lactate dehydrogenase (LDH), neutrophil-lymphocyte ratio (NLR), CRP, and APL-Abs.

## Materials and Methods

### Study Design and Participants

This study was a cross-sectional approach done from September 8 until November 4, 2020. All study participants had a confirmed case of COVID-19 and were hospitalized in Saiful Anwar General Hospital, Malang, Indonesia. A confirmed case was defined according to the case definition provided by WHO with positive results of SARS-Cov-2 from the respiratory specimen by real-time reverse transcription-polymerase chain reaction (RT-PCR) [16]. The ethical committee of Saiful Anwar General Hospital, Malang, Indonesia, approved this study (ethical number 400/194/K.3/302/2020). Oral consent was obtained from the patient or their relatives if the patients were unable to communicate. Subjects who got vitamin D supplementation in the last two years were excluded from the study.

### Data Collection

Data were obtained from the patient's medical records, including early signs and symptoms, underlying comorbidities, admission to intensive care unit (ICU), treatment, and outcome of the patient. Thrombotic events were defined as the presence of arterial or venous thromboembolism, such as deep vein thrombosis (DVT), acute coronary syndrome (ACS), cerebrovascular accident (CVA), and pulmonary embolism (PE). The appropriate examination evaluated subjects clinically suspected with thrombotic events to establish the diagnosis (compression ultrasound for DVT, head CT-scan for CVA, electrocardiography (ECG) and cardiac enzyme for ACS ECG, thorax plain radiograph, and CT-scan for PE).

Several routine laboratory examinations also measured at first-time patient admitted to the hospital, including haemoglobin, leukocyte, thrombocyte, prothrombin time (PT), activated partial thromboplastin time (APTT), D-dimer, fibrinogen, lactate dehy drogenase (LDH), alanine aminotransferase, aspartate aminotransferase, urea, and creatinine. Neutro phil lymphocyte ratio (NLR) was measured by the fraction of total neutrophil and lymphocyte count. C-reactive protein (CRP) and ferritin were measured by Chemiluminescent immunoassay (Cobas e411). The anti-phospholipid antibody, including IgM anti-beta2-glycoprotein and IgM anti-cardiolipin, were measured by ELISA (Orgentec Diagnostika GmbH). Serum vitamin D (25-Hydroxyvitamin D3) was obtained on the first-day patient admitted and measured by ELISA (Elabscience).

Vitamin D deficiency was defined by serum vitamin D levels below 49.92 nmol/L [17]. The ISTH criteria for DIC were used to assess the probability of overt DIC from the study participants. ISTH DIC scores ≥5 were classified as an overt DIC [18]. According to previous research regarding the probability of D-dimer in predicting the COVID-19 prognosis, we also grouped the subjects by D-dimer level. Ddimer >2 mg/mL was classified by a higher risk of developing thrombotic events [5].

### Statistical Analysis

Categorical variables were described as frequency rates and percentages. Continuous variables were described using mean, median, and interquartile range (IQR) values. Normally distributed data were compared using a t-test; otherwise, the Mann-Whitney test was used. The proportion for categorical variables was compared using the chi-square test and the Fisher exact test when data were limited. Bivariate and multivariate logistic regression models were used to explore the risk factor associated with coagulopathy. All statistical analyses were performed using SPSS version 17.0.

## Results

### Clinical and Laboratory Characteristics of the Subjects According to Vitamin D Levels

According to the inclusion and exclusion criteria, 50 COVID-19 patients were included in this study, from September 8 until November 4, 2020. There were 42 patients (84%) who had deficient levels of serum vitamin D (<49.92 nmol/L), while only eight patients (16%) had ≥49.92 nmol/L. Clinical comparison between the COVID-19 patients with normal to insufficient and deficient vitamin D is shown in Table 1. Both groups' mean age was similar, with a mean 58.5±13.5 and 52.5±14.5 years old (p=0.283). There was also no statistical difference in sex and comorbidi-ties between these two groups. According to the sign and symptoms presented at the first time patient came to the hospital, patient with deficient levels of vitamin D had a statistically higher frequency of fever compared to the other group (66.6% vs. 25%, p=0.047), yet no other differences were found in the other signs and symptoms between two groups.

**Table 1:** Clinical Characteristics of the Subjects According to Vitamin D Status

	Vitamin D ≥49.92 nmol/L (n=8)	Vitamin D <49.92 nmol/L (n=42)	p
Age (years) (mean ± SD)	58.5 ± 13.5	52.5 ± 14.5	0.283
Sex, n (%)			
Female	2 (25.0)	21 (50.0)	0.183
Male	6 (75.0)	21 (50.0)	
Sign and symptoms, n (%)			
Fever	2 (25.0)	28 (66.6)	0.047
Dry cough	5 (62.5)	27 (64.3)	0.923
Shortness of breath	5 (62.5)	26 (61.9)	0.975
Diarrhea	0 (0)	9 (21.4)	0.345
Nausea vomiting	1 (12.5)	14 (33.3)	0.239
Headache	1 (12.5)	9 (21.4)	0.563
Anosmia	2 (25.0)	8 (19.5)	0.700
Hemiparesis	0 (0)	3 (7.1)	0.436
Chest pain	0 (0)	4 (9.5)	0.363
Comorbidities, n (%)			
Diabetes mellitus	5 (62.5)	20 (47.6)	0.440
Hypertension	1 (12.5)	16 (38.1)	0.054
Malignancy	0 (0)	2 (4.8)	0.529
Cardiovascular disease	1 (12.5)	15 (35.7)	0.381
Chronic kidney disease	2 (25.0)	6 (14.3)	0.449
HIV infection	1 (25.0)	1 (2.4)	0.181
Admission, n (%)			
Non-ICU	8 (100)	34 (80.9)	0.080
ICU	0 (0)	8 (19.5)	
Thrombosis Manifestations, n (%)			
Acute coronary syndrome	0 (0)	2 (4.8)	0.529
Cerebrovascular accident	0 (0)	3 (7.1)	0.436
Deep vein thrombosis	0 (0)	1 (2.4)	0.659
In-Hospital Mortality, n (%)			
Survivor	8 (100)	33 (78.6)	0.148
Non-survivor	0 (0)	9 (21.4)	

There were eight patients admitted to the ICU because of respiratory failure and the need for a ventilator. All of them had low levels of vitamin D (<49.92 nmol/L); on the other hand, no patient was admitted to ICU from the group of patients with vitamin D levels < 49.92 nmol/L (p=0.080). We found 6 out of 50 patients (12%) had thrombosis manifestations: 2 (4%) had acute coronary syndrome, 3 (6%) had CVA thrombosis, and 1 (2%) had DVT. They were from the groups of patients with vitamin D levels <49.92 nmol/L (p=0.254). Among 50 subjects included in this study, nine patients (18%) died during treatment, and all of them had vitamin D levels <49.92 nmol/L.

Comparison of laboratory findings of patients according to the vitamin D levels was shown in Table 2. There was no statistical difference in the laboratory parameters from complete blood counts, liver, and renal function tests from both groups. Subjects with vitamin D levels <49.92 nmol/L had significantly higher D-dimer levels compared to the other group (median 4.4 (2.2-9.8) vs. 1.2 (0.8-2.6) mg FEU/L, p=0.001), but there is no difference in PPT, INR, APTT, or fibrinogen levels between these two groups. Patients with vitamin D levels <49.92 nmol/L also showed higher levels of inflammatory markers, such as CRP (median 4.6 (1.8-9.9) vs. 0.5 (0.3-0.7) mg/L, p=0.048) and Ferritin (median 8045.0 (4098.5-13334.5) vs. 2603.0 (1424.2-4499.2) mg/L, p=0.028) compared to the patients with vitamin D levels ≥49.92 nmol/L. However, no statistical difference was shown from LDH between the two groups. Five patients were found to be positive for APL-Abs: 1 (2%) patient had positive IgM anti-beta2 glycoprotein, and 4 (8%) patients had positive IgM anti-cardiolipin. All of them were from the groups of patients with vitamin D levels <49.92 nmol/L.

**Table 2:** Comparison of Laboratory Findings according to Vitamin D Levels

	Normal Value	Median (IQR)		p
		Vitamin D ≥49.92 nmol/L (n=8)	Vitamin D <49.92 nmol/L (n=42)	
Complete blood counts				
Hemoglobin (g/L)	134–177	135 (121–138)	128 (114–139)	0.397
Leukocyte (x10^9^/L)	4.3–10.3	7.6 (5.8–8.1)	10.8 (7.4–15.7)	0.948
Thrombocyte (x10^9^/L)	142–424	276 (251.5–351.0)	275.0 (236.0–329.0)	0.490
Neutrophil/Lymphocyte ratio (NLR)	<3.13	4.9 (3.8–5.2)	7.2 (5.5–10.2)	0.668
Hemostatic functions				
PPT (s)	9.4–11.3	10.8 (10.6–10.9)	10.9 (10.5–11.9)	0.990
INR	<1.5	1.0 (1.0–1.1)	1.1 (1.0–1.2)	0.990
APTT (s)	24.6–30.6	26.0 (25.1–29.6)	29.4 (25.5–33.9)	0.866
D-dimer (mg/L)	0.5	0.6 (0.4–1.3)	2.2 (1.1–4.8)	0.001
Fibrinogen (g/L)	4.54–11.70	7.61 (7.15–9.60)	8.85 (8.21–10.90)	0.490
Liver function test				
Alanine aminotransferase (U/L)	0–41	56.0 (37.5–69.0)	55.0 (31.0–99.0)	0.850
Aspartate aminotransferase (U/L)	0–40	29.0 (27.5–47.5)	28.5 (51.5–69.0)	1.000
Renal function test				
Urea (mmol/L)	2.7–8.0	2.8 (2.7–3.7)	5.5 (3.6–14.1)	0.140
Creatinine (mmol/L)	<0.106	0.097 (0.079–0.097)	0.088 (0.061–0.132)	0.698
eGFR (CKD-EPI) (mL/min/1.73 m^2^)	≥60	83.0 (73.5–90.0)	76.0 (40.5–96.5)	0.469
Inflammatory markers				
Lactate dehydrogenase (U/L)	240–480	373.0 (354.5–502.5)	707.5 (539.0–1039.0)	0.556
Ferritin (mg/L)	30–400	2603.0 (1424.2–4499.2)	8045.0 (4098.5–13334.5)	0.028
C-reactive protein (mg/L)	<3.0	5 (3–7)	46 (18–99)	0.048
Anti-Phospholipid Antibody, n (%)				
Positive IgM anti-beta2 glycoprotein	Negative if <5 U/mL	0 (0)	1 (2.4)	0.659
Positive IgM anti-cardiolipin	Negative if <7 U/mL	0 (0)	4 (9.5)	0.363

### Comparison of Vitamin D Levels According to Clinical Status of the COVID-19 Patients

**Figure 1:**
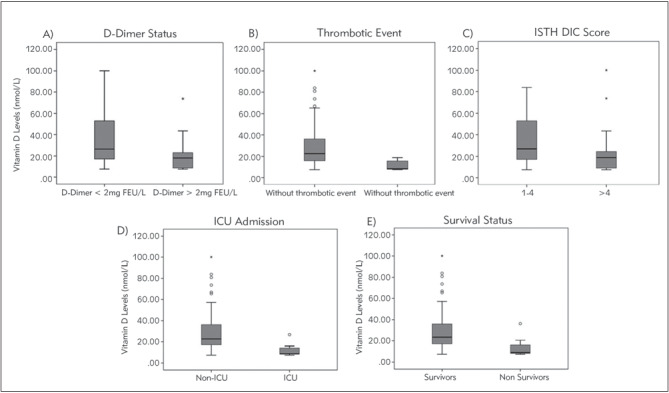
Comparison of Vitamin D Levels According to Clinical Status

Patients were grouped according to the severity of coagulopathy and COVID-19 (D-dimer >2 mg/L, the presence of thrombotic events, ISTH DIC score, ICU admission, and in-hospital mortality). Vitamin D level was compared between these grouped parameters (shown in Figure 1). Patient with D-dimer levels >2 mg/L had significantly lower vitamin D levels (median 18.22 (8.74-23.21) vs. 26.71 (16.97-53.91), p=0.006). The lower level of vitamin D also showed in the group of patients who developed thrombotic events (median 8.99 (7.74-18.72) vs. 22.21 (15.72–36.19); p=0.015). We also grouped these patients based on ISTH DIC score; overt DIC was categorized by ISTH DIC score ≥5 based on PPT, thrombocyte, D-dimer, and fibrinogen levels. We found that 29 patients (58%) had ISTH DIC score le 5 with significantly lower vitamin D levels (median 18.72 (8.99-24.46) vs. 26.96 (17.22-52.92), p=0.020) compared to the patients with ISTH DIC score 1-4. The patients admitted in ICU and those belonging to non-survivor groups of COVID-19 patients also consistently had significantly lower vitamin D levels.

### Risk Factors Associated with Coagulopathy among COVID-19 Patients

Clinical manifestations of coagulopathy in COVID-19 were presented as arterial or venous thrombosis as seen in our six patients. However, because of limitations of the numbers who developed the thrombosis, direct analysis for observing factors associated with thrombosis might be inconclusive. Therefore, we analysed the risk of COVID-19 coagulopathy by assessing the increased number of Ddimer (>2 mg/L) and ISTH score ≥5. Bivariate and multivariate model analysis for risk factors associated with increased D-dimer was seen in Table 3. In bivariate analysis, hypertension, urea >8.3 mmol/L, and vitamin D levels <49.92 nmol/L were associated with increased D-dimer levels. When these variables were included in the multivariate model analysis, only vitamin D levels were associated with D-dimer increase. The bivariate model for the ISTH DIC score was shown in Table 4, and only the vitamin D level was associated with ISTH DIC score ≥5. Because only one variable statistically significant, the multivariate analysis for the ISTH DIC score was not continued.

**Table 3:** Factors Affecting the Increase of D-Dimer (>2 mg/L) from Subjects

	Bivariate Analysis		Multivariate Analysis	
	OR (95% CI)	p	OR (95% CI)	p
Age ≥60 years old	1.3 (0.3–3.6)	0.832	-	-
Female	0.7 (0.2–2.2)	0.586	-	-
Diabetes mellitus	1.4 (0.5–4.2)	0.571	-	-
Hypertension	6.5 (1.7–24.6)	0.004	0.2 (0.1–1.1)	0.067
Cardiovascular disease	2.4 (0.7–8.1)	0.159	-	-
Chronic kidney disease	1.1 (0. –4.9)	0.902	-	-
Anemia	1.4 (0.4–4.2)	0.571	-	-
Leukocyte >12.0 x10^9^/L	2.3 (0.7–7.5)	0.164	-	-
Thrombocyte <150 x10^9^/L	6.6 (0.7–61.1)	0.056	-	-
NLR >3.13	0.4 (0.1–1.4)	0.138	-	-
INR >1.5	2.3 (0.2–26.8)	0.502	-	-
APTT ratio >1.3	1.6 (0.5–5.4)	0.423	-	-
ALT >41 U/L	1.3 (0.4–4.3)	0.641	-	-
AST >40 U/L	0.5 (0.2–1.7)	0.294	-	-
Urea >8.3 mmol/L	8.4 (1.9–75.1)	0.023	0.4 (0.1–3.2)	0.456
Creatinine >0.106 mmol/L	3.0 (0.8–11.4)	0.102	-	-
eGFR <60 mL/min/1.73 m^2^	3.2 (0.6–13.5)	0.103	-	-
LDH >480 U/L	0.7 (0.2–3.1)	0.634	-	-
Ferritin >400 µg/L	1.1 (0.2–4.9)	0.897	-	-
CRP >0.3 mg/L	0.8 (0.2–3.9)	0.826	-	-
Vitamin D <49.92 nmol/L	6.2 (1.1–34.2)	0.021	1.8 (1.2–4.4)	0.034
Positive IgM anti-cardiolipin	1.1 (0.1–8.4)	0.933	-	-

**Table 4:** Factors Affecting the ISTH DIC Score ≥5 among Subjects

	Bivariate Analysis	
	OR (95% CI)	p
Age ≥60 years old	1.2 (0.4–3.9)	0.738
Female	0.8 (0.3–2.5)	0.704
Diabetes mellitus	1.2 (0.4–3.6)	0.774
Hypertension	3.4 (0.8–12.8)	0.075
Cardiovascular disease	3.0 (0.8–11.2)	0.095
Chronic kidney disease	1.3 (0.3–5.9)	0.778
Anemia	1.6 (0.5–5.1)	0.567
Leukocyte > 12.0 x 10^9^/L	1.8 (0.5–5.9)	0.352
Thrombocyte <150 x10^9^/L	4.2 (0.4–38.7)	0.380
NLR >3.13	0.6 (0.2–2.4)	0.485
INR >1.5	0.5 (0.4–7.1)	0.128
APTT ratio >1.3	1.9 (0.6–6.8)	0.291
ALT >41 U/L	2.6 (0.8–8.8)	0.142
AST >40 U/L	0.8 (0.2–2.5)	0.668
Creatinine >0.106 mmol/L	3.7 (0.8–16.4)	0.101
eGFR <60 mL/min/1.73 m^2^	5.0 (0.9–27.4)	0.077
LDH >480 U/L	1.2 (0.3–5.6)	0.784
Ferritin >400 µg/L	1.8 (0.4–8.1)	0.425
CRP >0.3 mg/L	0.7 (0.1–3.4)	0.673
Vitamin D <49.92 nmol/L	5.4 (1.0–30.2)	0.039
Positive IgM anti-cardiolipin	2.3 (0.2–23.9)	0.473

### Correlation of Vitamin D Levels with Other Inflammatory Markers in COVID-19 Patients

**Figure 2:**
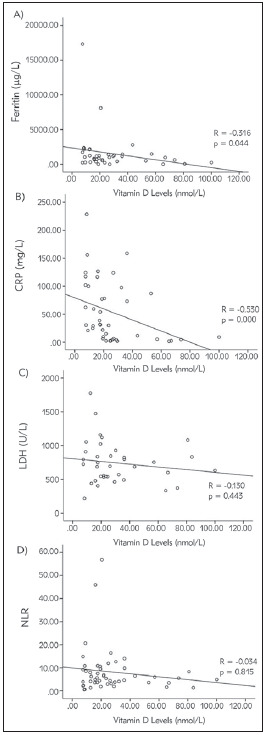
Correlation between Vitamin D Levels and other Inflammatory Markers

To observe the possible mechanisms of low vitamin D levels among COVID-19 patients, the correlation analysis between serum vitamin D levels with other inflammatory markers, such as CRP, ferritin, LDH, and NLR, was also done. Figure 2 showed the correlation analysis between these variables. Ferritin and CRP levels were negatively correlated with vitamin D levels (R=-0.316, p=0.044, and R=-0.530, p=0.000, respectively). On the other hand, LDH and NLR were not correlated statistically (R=-0.130, p=0.443, and R=-0.034, p=0.815, respectively).

## Discussion

The high mortality and its relationship with coagulopathy in COVID-19 have increasingly attracted attention. An increased incidence of arterial or venous thrombosis such as stroke and acute coronary syndromes has been reported in COVID-19 previously [19] [20]. In this study, among 50 patients confirmed with COVID-19, 12% of patients developed thrombosis, including DVT, stroke, and acute coronary syndrome. Several mechanisms of COVID-19 coagulopathy have been proposed; one that is exclusively being discussed is the role of vitamin D. Our present study showed that 84% of hospitalized patients with COVID-19 had vitamin D deficiency (<49.92 nmol/L). Low serum vitamin D levels have also been reported in other viral diseases, such as HIV and hepatitis infections [21] [22]. Recently, Radujkovic et al. [23] similarly showed that 64% of patients of COVID-19 had vitamin D levels <49.92 nmol/L and were associated with poor prognosis. Carpargano et al. [24] also observed that 81% of COVID-19 patients with acute respiratory failure treated in ICU had hypovitaminosis D.

In our report, a lower level of vitamin D was found in COVID-19 patients with thrombotic events, increased levels of D-dimer, and patients with overt DIC showed by the ISTH DIC score. Vitamin D level <49.92 nmol/L was also associated with elevated Ddimer level (>2 mg/L). ISTH DIC score < 5 indicated that low vitamin D level was an independent risk factor for the coagulopathy in COVID-19. The role of vitamin D on the coagulation pathway has been documented before. Khademvatani et al [13]. showed that lower vitamin D level was associated with idiopathic lower-extremity DVT. In a population-based study in Iran, low vitamin D levels were detected in a patient with venous thromboembolism [25]. The antithrombotic properties of vitamin D were believed to be through the activation of VDR. VDR knockout mice exhibited an enhanced platelet aggregation and promoted tissue thrombosis [12]. Another study also showed that vitamin D could improve endothelial function and reduce the production of proinflammatory cytokines, leading to inhibition of platelet aggregation [26].

The mechanisms of low vitamin D in COVID-19 is still not exact. Lack of published evidence contributes to the difficulty of answering this question. We found that vitamin D level was correlated with the inflammation markers CRP and ferritin. CRP and ferritin are acute phase reactants that have been established to be the biomarkers of hyper inflammation condition in COVID-19 [27] [28]. The correlation of vitamin D with these markers may explain that inflammatory conditions in COVID-19 can be attributed to the low level of vitamin D in these patients. Hyperinflammation in COVID-19, also described as a cytokine storm, is characterized by increased proinflammatory cytokines, such as IL-6 and TNF-α [29].

In another study, Xiong et al. [30] described TNF-α inhibiting the VDR expression in a dose and time-dependent manner on cultured proximal tubular epithelial cells. Meanwhile, Manion et al. [31] showed that deficiency of vitamin D among HIV patients was associated with high IL-6 levels similar to COVID-19.

Although several reports have shown the relationship between vitamin D and coagulopathy, there is still a lack of published data regarding the connection between vitamin D and coagulopathy in COVID-19. Besides, there is still a lack of guideline that recommends using vitamin D in COVID-19 until now. The clinical trial for the use of vitamin D supplementation in COVID-19 patients is also still ongoing research. Therefore, this study might help to be a preliminary study to provide a novel insight into the role of vitamin D in the COVID-19 coagulopathy. Our research is still limited to the small population size and restricted only to hospitalized patients. In the future, a multi-centre study with a larger size number or a population-based study can be performed to provide a better result.

In conclusion, we found that most hospitalized patients with confirmed COVID-19 had hypovitaminosis D. Deficient levels of vitamin D were independently associated with coagulopathy in COVID-19 patients. Low vitamin D levels might be associated with a hyperinflammatory condition in COVID-19 patients. The role of vitamin D deficiency in COVID-19 coagulopathy should be further evaluated and considered as a possible target for intervention.

*Acknowledgements*. The authors thank all investigators of participating clinicians in Saiful Anwar General Hospital, Malang, Indonesia, who helped in collecting the samples. This study was also supported by the Institute of Research and Community Services, University of Brawijaya by funding this research via Research and Management Development Grants in 2020.

## Conflict of interest statement

All the authors declare that they have no conflict of interest in this work.
